# Evidence for adaptive evolution of low-temperature stress response genes in a Pooideae grass ancestor

**DOI:** 10.1111/nph.12337

**Published:** 2013-05-23

**Authors:** Magnus D Vigeland, Manuel Spannagl, Torben Asp, Cristiana Paina, Heidi Rudi, Odd-Arne Rognli, Siri Fjellheim, Simen R Sandve

**Affiliations:** 1 Department of Medical Genetics, Oslo University Hospital and University of OsloOslo, Norway; 2 Helmholtz Zentrum München, Institute of Bioinformatics and Systems BiologyIngolstädter Landstrasse 1, München, Germany; 3 Department of Molecular Biology and Genetics, Aarhus UniversityDK-4200, Slagelse, Denmark; 4 Department of Plant and Environmental Sciences, Norwegian University of Life SciencesNO-1432, Ås, Norway

**Keywords:** adaptive evolution, climate adaptation, cold, habitat shift, Pooideae, temperate grasses

## Abstract

- Adaptation to temperate environments is common in the grass subfamily Pooideae, suggesting an ancestral origin of cold climate adaptation. Here, we investigated substitution rates of genes involved in low-temperature-induced (LTI) stress responses to test the hypothesis that adaptive molecular evolution of LTI pathway genes was important for Pooideae evolution.
- Substitution rates and signatures of positive selection were analyzed using 4330 gene trees including three warm climate-adapted species (maize (*Zea mays*), sorghum (*Sorghum bicolor*), and rice (*Oryza sativa*)) and five temperate Pooideae species (*Brachypodium distachyon*, wheat (*Triticum aestivum*), barley (*Hordeum vulgare*), *Lolium perenne* and *Festuca pratensis*).
- Nonsynonymous substitution rate differences between Pooideae and warm habitat-adapted species were elevated in LTI trees compared with all trees. Furthermore, signatures of positive selection were significantly stronger in LTI trees after the rice and Pooideae split but before the *Brachypodium* divergence (*P *< 0.05). Genome-wide heterogeneity in substitution rates was also observed, reflecting divergent genome evolution processes within these grasses.
- Our results provide evidence for a link between adaptation to cold habitats and adaptive evolution of LTI stress responses in early Pooideae evolution and shed light on a poorly understood chapter in the evolutionary history of some of the world's most important temperate crops.

## Introduction

The grass family (Poaceae) consists of *c*. 10 000 species, most of which belong to two major clades: BEP (Bambusoideae, Ehrhartoideae and Pooideae) (Soreng *et al*., 43) and PACCMAD (Panicoideae, Arundinoideae, Centothecoideae, Chloridoideae, Micrairoideae, Aristidoideae and Danthonioideae) (Gabriel Sánchez-Ken *et al*., 8). Poaceae evolved > 70 million yr ago in an ecosystem characterized by a warm climate (Grass Phylogeny Working Group, 11; Edwards & Smith, 6; Strömberg, 44), but have successively diversified outside their ecological zone of origin, and today we find grasses adapted to a wide range of climatic regimes, from tropical forests to freezing Arctic and Antarctic ecosystems.

**Figure 1:**
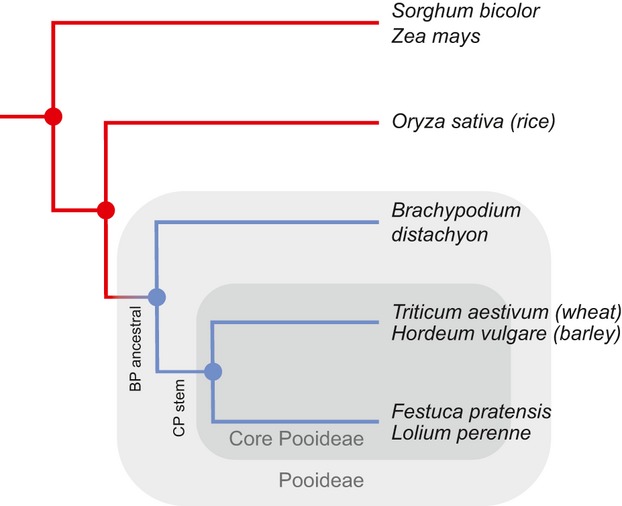
Study species and climate adaptation. Branch colors reflect subfamily differences in the mean temperature of the coldest month as shown in fig. S1 in Edwards & Smith (6). Hordeeae (*Triticum aestivum* and *Hordeum vulgare*), Poeae (*Festuca pratensis* and *Lolium perenne*), and PACMAD outgroup (*Sorghum bicolor* and *Zea mays*) clades are collapsed into single branches.

Pooideae, one of the most species-rich grass subfamilies, have successfully adapted to and diversified in cool climate ecosystems (Hartley, 15; Edwards & Smith, 6). However, it is unknown if cold and freezing tolerance evolved before, coincidentally with or during Pooideae evolution. What is clear is that Pooideae presently occupy much colder temperatures during the coldest month than other BEP lineages (see fig. S1 in Edwards & Smith, 6), suggesting a shift in climate adaptation some time between the BEP divergence (Fig. 1) and early Pooideae diversification. Pooideae consist of 14 tribes (Soreng *et al*., 43). The 10 earliest diverging, referred to hereafter as basal Pooideae (BP), represent a paraphyletic set of lineages with variable chromosome numbers (e.g. five, nine, 11 or 13) and significant morphological and ecological diversity, but only moderate species diversity, accounting for *c*. 20–30% of the total *c*. 3000 Pooideae species (http://delta-intkey.com) (Grass Phylogeny Working Group, 11; Hilu, 17). The earliest diverging BP tribe is Brachyelytreae, while the most recently diverging (i.e. sister to core Pooideae (CP)) is Brachypodieae. All four remaining tribes belong to a species-rich clade in which the basal chromosome number is seven, referred to as the core Pooideae (CP) (Fig. 1) (Soreng *et al*., 43; Hilu, 17). The CP encompass Hordeeae, Bromeae, and Poeae, in which all agriculturally important Pooideae crops belong.

How Pooideae evolved to become a highly successful cold-adapted lineage is not well understood. Comparative genomics has identified some intriguing examples of Pooideae-specific evolution of low-temperature-induced (LTI) responses involved in freezing tolerance. For example, the important C-repeat binding factor (CBF) gene family have diversified extensively in Pooideae (Skinner *et al*., 41; Li *et al*., 26) possibly through some selection-driven mechanism (Sandve & Fjellheim, 38). Two other intriguing examples are the Pooideae lineage gains of fructosyl transferase enzymes (Francki *et al*., 7; Li *et al*., 26) and the ice-interacting ice recrystallization inhibition proteins (Sidebottom *et al*., 40; Sandve *et al*., 37), which have both been shown to increase plant freezing tolerance (Hisano *et al*., 18; Zhang *et al*., 52). Although such fixations of lineage-specific molecular features may be driven by adaptive evolution, the existence of lineage-specific LTI stress responses *per se* reveals nothing about ancestral selection pressures. Moreover, these comparative genomics case studies do not provide a framework for testing hypotheses about the mechanisms involved in Pooideae cold climate adaptation.

One way to specifically test hypotheses concerning adaptive evolution is to reconstruct ancestral selection pressures by estimating the relationship between synonymous (dS) and nonsynonymous (dN) substitution rates (dN/dS) at individual codons during gene evolution (Zhang *et al*., 53). Branches with a dN/dS ratio > 1 are considered to reflect positive selection pressure (i.e. adaptive evolution), while dN/dS = 1 and dN/dS < 1 are signatures of neutral and purifying selection, respectively. If adaptive evolution was important for Pooideae cold climate adaptation, we predict stronger signatures of positive selection (elevated dN/dS ratio) in LTI genes compared with the genome-wide background level.

A simple model of Pooideae climate adaptation can be hypothesized on the basis of the evolution of habitat temperature preference in the BEP clade (Edwards & Smith, 6). According to this hypothesis, some change in the environment triggered adaptive evolution of cold-stress tolerance, and was followed by niche expansion and ecological diversification in cooler habitats, either before Pooideae divergence or during the evolution and diversification of Pooideae. This study is the first to reconstruct the evolution of substitution rates and ancestral selection pressure between early BEP diversification (the Ehrhartoideae–Pooideae split) and the diversification of the CP lineages, and specifically investigate whether LTI genes were key targets for adaptive evolution. Our results suggest that LTI genes were under strong adaptive selection before the divergence of the CP clade and reveal new insights into a poorly understood chapter in the evolutionary history of some of the world's most important crops.

## Materials and Methods

### Sequence data sets

Our choice of species to include was based on a balance between the availability of cDNA resources and taxonomic diversity. We included four sequenced genomes: those of sorghum (refers to the species *Sorghum bicolor*) and maize (*Zea mays*) as PACMAD outgroups, and the two sequenced BEP genomes of rice (*Oryza sativa*) and *Brachypodium distachyon*. No completely sequenced CP genome is available yet, and hence we selected species with large sequence resources distributed across two different CP clades: wheat (*Triticum aestivum*) and barley (*Hordeum vulgare*) belonging to the Hordeeae, and *Lolium perenne* and *Festuca pratensis* belonging to Poaeae. We did not include any Bambusoideae species because of the extremely long generation time (which influence substitution rates) and ambiguous placement in the BEP clade (Grass Phylogeny Working Group, 11; Grass Phylogeny Working Group II, 12). Annotations of coding sequences (CDSs) and proteins from the sequenced genomes were downloaded from ftp.plantbiology.msu.edu (Rice v6.1), ftp.brachypodium.org (Brachypodium v1.2), ftp.maizesequence.org (Maize v5a), and ftp://ftpmips.helmholtz-muenchen.de/plants/sorghum/ (Sorghum v1.4), respectively. CDS from the nonsequenced genomes of barley, wheat, *L. perenne*, and *F. pratensis* were compiled from a variety of sources. Barley nonredundant full-length cDNA sequences representing 23 588 genes (Mayer *et al*., 28) were kindly provided by Klaus Mayer. Wheat CDSs were compiled from a combination of transcripts from the unigene collection at the National Center for Biotechnology Information (NCBI; http://www.ncbi.nlm.nih.gov/unigene), putative unique transcripts (PUTs) were downloaded from the plant genome database (plantgdb.org), and a collection of full-length wheat cDNAs were downloaded from the Hordeeae full-length CDS DataBase (http://trifldb.psc.riken.jp/index.pl). The *F. pratensis* CDS data were compiled from PUTs (plantgdb.org) and an in-house collection of *de novo* assembled cDNA transcripts sequenced on the 454 Life Sciences (Branford, CT, USA) sequencing platform (Supporting Information Methods S1; NCBI sequence read archive accession numbers: SRX248127, SRX248124, SRX248128 and SRX248126). CDSs from *L. perenne* were *de novo* assembled from Illumina (San Diego, CA, USA) RNAseq data (Methods S2; raw data are deposited under the project title ‘Transcripts upregulated by cold treatment in *Lolium perenne* leaves’ in EMBL-EBI ArrayExpress).

Prediction of open reading frames (ORFs) in CDS collections from nonsequenced genomes was carried out in a pipeline together with redundancy removal and data cleaning. Homology-based ORF prediction was performed with OrfPredictor (Min *et al*., 29) using BLASTX results against annotated proteins from rice, *B. distachyon*, maize, and sorghum (e-value < 1e-5) as a guide. Redundancy in the data sets was subsequently removed using CD-HIT (Swindell, 46) with the parameters −c 0.99 –*n* 5, that is, a 99% sequence identity cut-off and a word size of 5. Finally, CDS sequence sets were filtered using functions in the *seqinr* package (Charif & Lobry, 4) in R (R Development Core Team, 35) to only contain proteins that start with a start codon (M), with a minimum length of 30 amino acids (aa), only containing unambiguous codons.

### Automated multiple sequence alignments and phylogenetic tree estimation

A pipeline for automated generation of gene sequence alignments and trees was scripted in R language. Initially identification of putative orthologous sequences was performed by identification of best reciprocal (BR) BLASTP to each *B. distachyon* gene. The BR-BLASTP was carried out as follows: for each species ‘S’, BLASTP identified the best hit between *B. distachyon*–‘ S ’ and ‘S’–*B. distachyon*. If the best hit was the same in both directions, we classified the gene from species ‘S’ as a putative ortholog of the *B. distachyon* gene. No filtering for BLAST hit scores was undertaken in this step, as our pipeline has stringent downstream filtering criteria. Next, the pipeline took orthologous sequence sets as identified by the BR-BLASTP as input data. Guidance (Penn *et al*., 34) was used to produce codon alignments for each orthologous sequence set. Our in-house script works in an iterative fashion to filter both poorly aligned sites and poorly aligned gene sequences as predicted by Guidance. For each alignment, any gene sequence with a Guidance score of < 0.90 was removed from the sequence set and Guidance was re-run. When all remaining gene sequences passed the sequence score cut-off, poorly aligned codons (Guidance column scores < 0.93) were removed from the alignment. From the resulting trimmed alignment, a maximum likelihood (ML) tree was estimated with the R package *phangorn* (Schliep, 39) using the general GTR + G + Γ model. Finally, two quality control tests were used to identify and remove trees containing errors or unreliable orthologous sequences. First, any ML tree whose tree topology deviated from the assumed true species topology (Fig. 1) was disregarded. Secondly, topologies were scanned for very long branch lengths (i.e. indicating inclusion of nonorthologous sequences) by checking whether the longest branch was more than three times longer than the second longest branch. If an external branch was identified as a long branch, the corresponding gene sequence was removed from the alignment, and the reduced sequence set was sent back to the initial Guidance pipeline step. Any topology with an internal long branch was discarded from further analyses. All scripts used in this pipeline are freely available upon request.

### Identification of low-temperature-induced genes

LTI trees were defined as having at least one LTI gene from either barley (Hordeeae) or *L. perenne* (Poeae). Barley LTI genes were predicted based on homology to 2778 unique barley Affymetrix (Santa Clara, CA, USA) microarray probes reported to be induced by low temperature (Svensson *et al*., 45; Tommasini *et al*., 47; Greenup *et al*., 13). The gene targets of the microarray probes were downloaded from affymetrix.com and were used as queries in BLASTX against the barley CDS predicted protein sequences. Only hits between probe targets and barley proteins with 100% identity were considered. Furthermore, only BLASTX hits that encompassed the full probe target sequence and covered > 90% of the target protein, or hits representing complete (± 5 aa) 5′ or 3′ exon sequences longer than 50 amino acids, were kept. Identification of *L. perenne* LTI CDSs was carried out using differential expression analyses between cold-treated and non-cold-treated plants (Methods S2). In short, transcript abundance was estimated using rsem (Li & Dewey, 25) and differential expression was identified using DESeq (Anders & Huber, 2). Expression data from wheat and *F. pratensis* were not used in this study because of difficulties of relating microarray probes to defined wheat orthologs in our study (possibly because of its hexaploid nature) and the lack of genome-wide expression studies available for *F. pratensis*.

### Estimation of molecular evolutionary rates and tests for positive selection

We used two methods of estimating evolutionary rates in genes of rice and Pooideae species relative to the outgroup species maize and sorghum. If both outgroup species were present in the same tree, the mean distance to these outgroups was used. First, ML-GTR evolutionary distances to outgroups were extracted directly from the trees using the cophenetic.phylo function in the *ape* R-package (Paradis *et al*., 33). The GTR tree distance is a function of the likelihood of observing different substitutions at individual nucleotide sites; hence this method does not take protein-level changes into account. As an alternative approach, we used paml (Yang, 50) to break down the evolutionary distances into estimates of synonymous (dS) and nonsynonymous (dN) substitution rates (codeml function, codon model, model 2). The rates dS and dN are defined as the number of synonymous/nonsynonymous substitutions per synonymous/nonsynonymous site. Rate estimates of dS > 2 and/or dN > 0.5 were considered as outliers and removed from further analyses. As GTR and paml use different underlying models of DNA sequence change, their estimates of evolutionary distance are not directly comparable.

The branch-site model (Zhang *et al*., 53) in codeml was used to test for positive selection along the ancestral BP and stem CP branches. The likelihood of data (alignment and tree) was evaluated by codeml under the two competing nested models: (1) codons only evolve under purifying selection (dN/dS < 1) or neutral selection (dN/dS = 1), or alternatively (2) codons are allowed to evolve under positive selection (dN/dS > 1) as well as under purifying and neutral selection. Each test was run applying four different starting values for dN/dS estimates (i.e. omega &= ω) for site classes under positive selection (0.5, 1, 1.5, and 2) and the results from the analyses having the highest likelihood score were used (Yang & dos Reis, 51). Separate codeml analyses were run allowing for positive selection on ancestral BP and CP stem branches (i.e. foreground branches) (Fig. 1). A likelihood ratio test (LRT) under a chi-square distribution (1 degree of freedom) was then used to test the null hypotheses of purifying/neutral selection pressure. Correction for multiple testing was performed using false discovery rate in the p.adjust function in R (R Development Core Team, 35).

### Resampling tests

Resampling tests were used to test for differences in test statistics distribution between random subsets of all trees with identical size as the LTI subset and the LTI subset. The resampling tests were programmed in R using the sample function, and *P*-values were calculated as the proportion of 50 000 resampled data sets with equal or more extreme test statistics as observed in the LTI trees.

### Gene family size prediction

OrthoMCL v2.0 (Li *et al.,* 500) was used to define gene family clusters to assess if there was any systematic bias in gene family size and hence also a possible bias in the purifying selection pressure. In a first step, pairwise sequence similarities between all input protein sequences were calculated using BLASTP with an e-value cut-off of 1e-05. Markov clustering of the resulting similarity matrix was used to define the ortholog cluster structure, using an inflation value (−I) of 1.5 (OrthoMCL default). Splice variants were removed from the data set, keeping the longest protein sequence prediction.

### Gene ontology over- and under-representation analysis

To be able to assess sample bias in gene function between the LTI subset and all genes, gene ontology (GO) terms, that is, a common set of hierarchical terms related to gene/protein function, were acquired for *B. distachyon* genes using InterproScan (Hunter *et al*., 20). Only GO terms from the category of molecular function were considered because of better transferability and comparability. To identify GO terms over- and under-represented in LTI genes versus all genes, we used the GOstats R package (Gentleman, 10) from Bioconductor (http://www.bioconductor.org/). Plant GO slim terms were extracted for each MF GO term using the web-service at agbase (http://agbase.msstate.edu/cgi-bin/tools/goslimviewer_select.pl).

## Results

### Reconstruction of orthologous gene trees

CDS prediction of wheat, *F. pratensis*, and *L. perenne* transcripts resulted in data sets of 32 440, 25 998, and 43 049 CDSs, respectively. Initial ortholog clustering produced 6479 putative orthologous CDS sets containing at least one species from each of Hordeeae (wheat/barley) and Poaeae (*L. perenne/F. pratensis*), as well as CDSs from *B. distachyon*, rice, and at least one of the two PACMAD outgroup species. A total of 4330 of the orthologous sequence sets (Notes S1, S2) produced trees that passed the filtering criteria in our automated alignment and phylogeny estimation pipeline (Table 1). The median number of species per tree was seven, with 128, 1199, 2095, and 908 trees containing five, six, seven, and eight taxa, respectively. A total of 388 trees (8.9%) were classified as LTI trees. The median number of species per LTI tree was also seven, with three, 90, 205, and 90 trees containing five, six, seven, and eight taxa, respectively. The mean number of aligned residues was also similar between all trees and the LTI subset (1244 and 1165 bp, respectively) (Table 1), indicating that the LTI subset has no major bias in alignment quality or taxon content.

**Table 1:** Summary statistics for orthologous sequence alignments

Species	All				Low temperature induced						
	No. CDS	Aligned base pairs (mean (range))	Total aligned (kb)	No. CDS	Aligned base pairs (mean (range))	Total aligned (kb)
*Brachypodium distachyon*	4330	1389 (195–7938)	6013	388	1277 (321**–**4410)	495
Barley	3771	1335 (201**–**5334)	5034	373	1245 (270**–**4398)	464
Wheat	3219	1050 (159**–**6738)	3381	300	1043 (207**–**3687)	313
*Lolium perenne*	4043	1161 (135**–**6771)	4693	360	1128 (231**–**4368)	406
*Festuca pratensis*	1780	889 (132**–**3075)	1582	152	786 (132**–**2109)	119
Rice	4330	1383 (183**–**7296)	5990	388	1273 (342**–**4404)	494
Maize	4088	1370 (171**–**7497)	5598	371	1283 (312**–**4410)	476
Sorghum	4202	1375 (186**–**7728)	5777	378	1287 (342**–**4410)	486

CDS, coding sequence.

As opposed to the alignment statistics, the LTI subset differed significantly from all trees in terms of distribution of GO molecular function classes. Nine GO terms were under-represented, and 33 over-represented in the LTI subset (Tables S1, S2). Among the under-represented categories were DNA binding and diverse kinase functions, while many of the over-represented GOs were related to oxidative stress response, protein translation, carbohydrate metabolism, lipid binding, glycolysis, and RNA binding.

### Substitution rates are increased in Pooideae relative to rice

**Figure 2:**
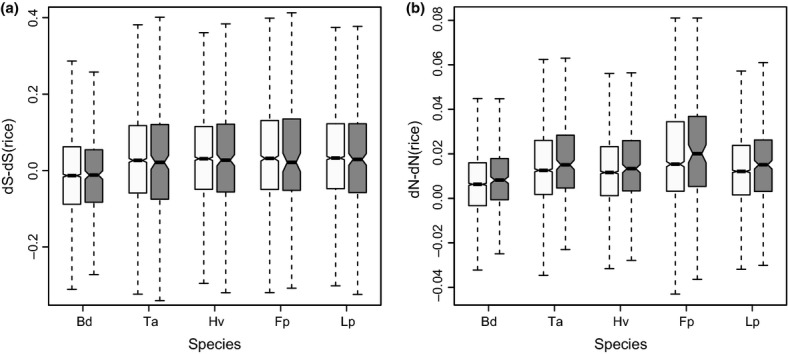
Difference in synonymous (dS) and nonsynonymous (dN) rates between Pooideae species and rice. (a) Intra-phylogeny dS difference between Pooideae species and rice. (b) Intra-phylogeny dN difference between Pooideae species and rice. White, all loci; gray, low-temperature-induced (LTI) loci. Outliers are excluded from the plot using the ‘outline = F’ option in the R boxplot function. Latin species names are abbreviated: Bd, *Brachypodium distachyon*; Ta, *Triticum aestivum* (wheat); Hv, *Hordeum vulgare* (barley); Lp, *Lolium perenne*; Fp, *Festuca pratensis*.

Our ML-GTR results show a clear trend of differences in substitution rates among rice, *B. distachyon* and CP species, with CP species > *B. distachyon* > rice (Table 2). To assess the contribution of synonymous and nonsynonymous substitutions to the rate differences, we further decomposed evolutionary rates into dS and dN using paml. All dS and dN estimates were higher in CP species compared with the rice lineage, but for *B. distachyon* only the dN was elevated compared with rice (Table 2, Fig. 2a,b). Hence, the substitution rates of the BEP species reflect two major patterns. First, there is a general trend of substitution rate increase in Pooideae relative to rice, and secondly, these rate differences are not homogenous within Pooideae. Identical trends of increased substitution rates in Pooideae relative to rice were observed in the LTI subset; however, the median distance to the PACMAD outgroup was slightly smaller compared with all trees (Table 2) (i.e. LTI genes evolved at a lower rate).

**Table 2:** Median evolutionary distances to the outgroup species *Sorghum bicolor* and *Zea mays* as estimated by the general time reversible (GTR) model, and synonymous (dS) and nonsynonymous (dN) substitutions per synonymous/nonsynonymous site

Trees	Method	Rice	Pooideae							
			Bd	Hv	Ta	Fp	Lp
All	GTR	0.220	0.230	0.250	0.253	0.262	0.253
	dS	0.638	0.619	0.661	0.665	0.657	0.672
	dN	0.071	0.077	0.083	0.082	0.091	0.083
Low temperature induced	GTR	0.206	0.224	0.244	0.246	0.267	0.252
	dS	0.641	0.624	0.657	0.669	0.667	0.686
	dN	0.063	0.070	0.077	0.075	0.080	0.077

Bd, *Brachypodium distachyon*; Hv, *Hordeum vulgare* (barley); Ta, *Triticum aestivum* (wheat); Fp, *Festuca pratensis*; Lp, *Lolium perenne*.

### LTI trees show elevated dN for Pooideae species

The difference between rice and Pooideae dN was significantly increased in LTI trees relative to all trees (Table 3, Fig. 2b), but no such pattern was observed for the dS estimates (Fig. 2a). Elevated Pooideae dN in the LTI tree subset could theoretically be caused by a relative decrease in rice dN for these genes. We therefore estimated the dN difference between maize–rice and sorghum–rice using the core Pooideae species as outgroups, but no difference between the LTI tree subset and all trees was observed in these analyses (Fig. S1). This supports a scenario of increased dN/dS ratio for LTI genes in the Pooideae lineage after the split from Ehrhartoideae, which could be caused by increased positive selection or, alternatively, relaxation of purifying selection pressure in Pooideae relative to rice.

**Table 3:** Observed and resampled rate differences for low-temperature-induced (LTI) orthologs

Comparison	Method	*Brachypodium distachyon*–rice				Core Pooideae–rice					
		Observed (LTI)	Resampled	Observed (LTI)	Resampled
LTI versus all	GTR	0.009	0.009	0.036*	0.032
	dS	−0.011	−0.013	0.033	0.036
	dN	0.008*	0.006	0.016**	0.014

The core Pooideae estimate is the mean across all orthologs from core Pooideae species.

Resampled rate differences shown are the mean over 50 000 resampled medians.

GTR, general time reversible; dS, synonymous substitutions per synonymous site; dN, nonsynonymous substitutions per nonsynonymous site.

* *P *< 0.05;

** *P *< 0.01.

Bias in gene family size could result in different purifying selection pressures. Gene duplication generates redundant gene copies and commonly results in relaxation of purifying selection pressure in one or both of the copies. We therefore estimated gene family size for each locus to make sure that Pooideae LTI genes were not biased toward larger family sizes. Mean gene family sizes were 1.44, 1.31, and 1.31 for barley, *B. distachyon*, and rice, respectively. For genes in the LTI tree subset, the gene family size estimates were 1.63 for barley, 1.52 for *B. distachyon*, and 1.51 for rice, suggesting that genes in LTI genes belong to slightly larger gene families than the average gene. However, the difference in gene family size between rice and the Pooideae species was not larger for LTI trees compared with all trees.

### Tests for positive selection on BP and CP branches

Tests for positive selection using the branch-site model in paml were conducted to compare the magnitude of ancestral positive selection pressure on Pooideae genes in the LTI subset relative to all genes. If LTI genes experienced stronger positive selection pressure compared with the average gene during Pooideae evolution, we would expect likelihood ratio distributions biased upward, and proportionally more significant tests on branches in LTI trees compared with all trees. Nine (2.3%) (Table 4) of the LTI trees had a significant LRT for positive selection on the BP ancestral branch, a significantly higher proportion compared with all trees (28 loci; 0.6%) (resampling *P*-value = 0.0495). A similar test on the stem branch of the CP clade produced a lower proportion of significant tests for positive selection in LTI trees (one locus; 0.3%) compared with all trees (18 loci; 0.4%). Resampling tests for larger likelihood ratios in LTI trees (using the third quartile as a test statistic) were also significant for the BP ancestral branch (*P *= 0.0178), but not for the CP stem branch only (*P *= 0.33). All likelihood estimates from the codeml results are found in Tables S3 and S4.

**Table 4:** Low-temperature-induced genes under positive selection

Branch	Gene	Annotation	Putative function(s)	No selection (log likelihood)	Selection (log likelihood)	*P*-value (fdr)	Sites
BP	Bradi1 g13640.1	Chaperone J2	Co-chaperone activity	−2539.972	−2533.284	0.019	7, 231
	Bradi2 g38290.1	ku70-binding protein	Double strand break repair	−1855.013	−1849.220	0.028	116
	Bradi2 g55070.1	SOUL heme binding protein	Red/far-red light signaling	−1727.853	−1719.885	0.009	48, 183
	Bradi2 g58050.1	Fructose-bisphosphate aldolase	Glycolysis	−3248.868	−3242.741	0.022	82, 97, 285
	Bradi3 g17200.1	Tyrosyl-tRNA synthetase	Translation	−3324.640	−3316.783	0.009	21, 30, 157
	Bradi4 g09430.1	Acidic endochitinase	Disease response	−2495.464	−2486.970	0.009	38, 60, 167, 200, 205, 223, 226
	Bradi4 g34170.1	Ribosomal protein S16	Translation	−1177.237	−1170.983	0.022	101, 135
	Bradi4 g36800.1	Phospholipase D delta	Cell membrane lipid hydrolysis/signaling	−7418.390	−7411.998	0.022	87, 93, 150, 203, 250, 444, 819
	Bradi5 g25050.1	Naringenin 3-dioxygenase	Flavanoid biosynthesis	−2904.277	−2897.533	0.019	273, 293
CP	Bradi1 g35200.2	Novel plant SNARE 11	Membrane receptor/protein transport	−2251.089	−2241.895	0.007	72, 113, 137

Annotations of putative functions are based on *Brachypodium distachyon* gene homology to *Arabidopsis thaliana* proteins.

BP, basal Pooideae ancestral; CP, core Pooideae stem.

Sites refer to codons in the trimmed alignments with a Bayes empirical Bayes posterior probability ≥ 0.9. All point estimates for foreground omega values (ω2) were > 7.

## Discussion

### A link between cold habitat adaptation and adaptive evolution in ancestral Pooideae

Pooideae is recognized as a group with cool climate adaptations (Grass Phylogeny Working Group, 11; Edwards & Smith, 6), but little is known about the molecular evolution involved in turning the Pooideae lineage temperate. In this study we demonstrate that the difference in dN rates between Pooideae species and grasses that are not adapted to cold ecosystems is significantly increased in LTI trees compared with all trees (Fig. 2, Table 3). Moreover, this dN difference cannot be explained by relaxation of selection pressure as a result of bias in gene family size and functional redundancy. We also demonstrate a significantly higher proportion of trees with signatures of positive selection before the CP diversification in the LTI subset compared with all trees (Fig. 1, Table 2). Unsurprisingly, GO analyses showed significant differences between the LTI genes and the total gene set, and it is conceivable that this might have biased our comparisons of positive selection signatures. However, the substitution rate estimates are not GO-biased, as we compared rates within trees, and these agree well with the tests for positive selection. Taken together, our results support strong positive selection on LTI genes between the Ehrhartoideae–Pooideae and *B. distachyon*-CP splits, and represent evidence for a link between the ancestral habitat shift in the BEP clade (Edwards & Smith, 6) and adaptive molecular evolution. Generation of additional genomic resources from species in more basal Pooideae lineages is needed to be able to precisely pinpoint the timing of changes in selection pressure on LTI genes and better understand the interconnection between climate adaptation and BEP clade evolution.

Early Pooideae evolution has also been suggested to be associated with enhanced drought stress tolerance, as ancestral Pooideae moved out of forests and into open habitats (Kellogg, 21); however, it is also feasible that open habitats also were characterized by being cooler. Stress recognition and signal transduction pathways involved in low-temperature and drought stresses overlap substantially (Swindell, 46; Yamaguchi-Shinozaki & Shinozaki, 49), and hence it is challenging to disentangle ancestral selection pressures caused by the two environmental factors. Published drought stress expression data (Guo *et al*., 14; Abebe *et al*., 1) only enabled us to define 22 trees containing drought-induced genes in our data set (nine genes were contained in the LTI subset; data not shown), and we were thus not able to make comparable analyses with LTI genes. Detailed transcriptome maps of stress-specific responses are needed to be able to investigate whether adaptive evolution was stronger in drought or low-temperature stress response pathways during BEP evolution and diversification.

Inferences about the causality of specific molecular changes and improved cold climate adaptation in this study will be speculative in nature. Nevertheless, a few anecdotal observations are worth mentioning. Firstly, one of the LTI orthologs that evolved under positive selection is homologous to phospholipase D-δ, an essential enzyme for freezing tolerance in Arabidopsis through its involvement in lipid hydrolysis and signaling (Li *et al*., 27). Secondly, four other LTI genes under positive selection have predicted molecular functions known to be important in responses to cold and freezing stress: antioxidant activity (Bradi5 g25050.1) (Kovtun *et al*., 22; Mittler *et al*., 30), protein translation (Bradi3 g17200.1 and Bradi4 g34170.1) (Nakaminami *et al*., 31; Rogalski *et al*., 36), and glycolysis (Bradi2 g58050.1) (Conley *et al*., 5; Hashimoto & Komatsu, 16; Soitamo *et al*., 42). The identification of these low-temperature stress-related genes verifies our approach and provides interesting targets for further research on the evolution of low-temperature stress responses in grasses.

### Genome-wide substitution rate heterogeneity in Ehrhartoideae and Pooideae

In addition to strong signatures of positive selection on Pooideae LTI genes, we also observed a genome-wide trend of heterogeneous substitution rates in the BEP clade (Figs 2). Such rate differences can be caused by differences in reproductive systems (i.e. generation time or inbreeding versus outbreeding), population history (e.g. bottlenecks or population expansions), or mutation rate, or changes in selection pressure (reviewed in Gaut *et al*., 9). These factors are, however, unlikely explanations of our results as the species in our study are a mix of facultative inbreeders (rice, *B. distachyon*, wheat, and barley) and obligate outbreeders (*F. pratensis* and *L. perenne*) with negligible differences in generation times. Technical artifacts from sequencing and assembly errors can affect substitution rate estimates but as the CDS sequences were generated from a variety of sequencing platforms and assembly software (see methods) this is unlikely to have had a systematic influence on our results.

Both *B. distachyon* and CP species have a genome-wide increase in dN compared with rice (Fig. 2, Table 2). This is a trademark signature of a population bottleneck; increased fixation of slightly deleterious nonsynonymous mutations as a result of a shift in the selection–drift balance (Ohta, 32). A more controversial explanation is adaptive evolution through the plasticity-relaxation-mutation (PRM) model (Hughes, 19). In the PRM model, initial phenotypic responses to a changing environment occur through plasticity, which is followed by relaxation of purifying selection pressure on genes affecting the ‘old’ phenotype which is no longer expressed. A dramatic switch in habitat could thus initiate relaxation of purifying selection pressure across hundreds or thousands of genes because of the multigenic nature of climate adaptation phenotypes.

In addition to the elevated dN compared with rice, CP species (but not *B. distachyon*) also have significantly higher dS (Fig. 2a, Table 2). Assuming no difference in reproductive biology, this must be caused by higher mutation rates. Several studies have identified a strong link between rates of speciation and the speed of molecular evolution (i.e. substitution rates) in both plants and animals (Barraclough & Savolainen, 3; Lanfear *et al*., 24; Venditti & Pagel, 48). This is consistent with the CP clade having undergone extensive speciation, and it presently contains *c*. 70–80% of all Pooideae species (http://delta-intkey.com). Other factors influencing dN and dS in the CP species could be elevated environmental stress (Lamb *et al*., 23) (i.e. in cooler climates) or higher genome redundancy in CP species compared with rice and *B. distachyon* (Barraclough & Savolainen, 3).

### Conclusions

This is the first set of genome-wide analyses that offers evidence of a link between adaptation to cold climates and adaptive evolution at the molecular level during the evolution of the major temperate grass clade Pooideae. Our results support an evolutionary model in which strong selection for novel and favorable molecular variants of LTI pathway genes enabled niche range expansion in cold climate ecosystems. However, further genomic resources are needed to pinpoint when selection pressure on LTI genes changed during BEP diversification and if genes involved in cold- or drought-induced response pathways have been under divergent or similar selection pressures.

## Supporting Information

**Methods S1** Sequencing and assembly of the Festuca pratensis transcriptome. **Methods S2** Lolium perenne transcriptome sequencing, assembly, and analysis of differential expression between plants with and without low-temperature treatment. **Table S1** Over-represented molecular function gene ontology (GO) terms in the subset of low-temperature-induced genes **Table S2** Under-represented molecular function gene ontology (GO) terms in the subset of low-temperature-induced genes

Notes S1 All orthologous sequence sets in raw form as R-list objects.

Notes S2 LTI orthologous sequence sets in raw form as R-list objects.

Table S3 Codeml results for the branch-site test on the most recent common ancestor of basal and core Pooideae (BP ancestral)

Table S4 Codeml results for the branch-site test on the most recent common ancestor of core Pooideae (CP stem)
