# Supplementary material for: Evidence for adaptive evolution of low-temperature stress response genes in a Pooideae grass ancestor
Source: New Phytol. 2013 May 23;199(4):1060–8. doi: 10.1111/nph.12337 (PMC3840698; doi:10.1111/nph.12337)
Supplement: Supplementary file 1 — Methods S1 Sequencing and assembly of the Festuca pratensis transcriptome. Methods S2 Lolium perenne transcriptome sequencing, assembly, and analysis of differential expression between plants with and without low-temperature treatment. Table S1 Over-represented molecular function gene ontology (GO) terms in the subset of low-temperature-induced genes Table S2 Under-represented molecular function gene ontology (GO) terms in the subset of low-temperature-induced genes [file nph0199-1060-sd1.pdf]

Methods S1

Sequencing and assembly of a *Festuca pratensis* transcriptome

Plant material and cDNA library construction

For the *F. pratensis* transcriptome sequencing we used two unrelated genotypes, a Yugoslavian cultivar (B14/16) and a Norwegian cultivar (HF2/7) (Ergon, Fang et al. 2006). Plants were grown at 17/12°C day/night, 16/8h light/darkness with light intensity of 135 µmolm^{-2}s^{-1} (400W HQI-BT Osram bulbs). Tissues from crown, stem and leaves were pooled and total RNA (100mg) was isolated using the RNeasy Mini kit (Qiagen, Hilden, Germany) and RNeasy MinElute kit (Qiagen).

Two microgram RNA was used for cDNA synthesis using MINT-Universal cDNA synthesis kit, protocol-II (Evrogen). Amplified cDNAs were purified using Qiaquick PCR purification kit (Qiagen), EtOH precipitated, air dried, dissolved in 20 micro liter of sterile water, and stored at -20°C until used for 454 sequencing on the GS-FLEX following the protocol of the manufacturer.

De novo sequence assembly

De novo assembly of the 454 transcriptome sequences was done with the CLC genomics workbench (http://www.clcbio.com/index.php?id=1240). Because our transcriptome sequences were derived from two highly heterozygous (out breeding) individuals, we used multi-step approaches in the process of creating de novo assembled cDNA contigs. In the first step, we

performed *de novo* assemblies using reads from one genotype at the time. Because grass genomes typically are complex in respect to paralog numbers we chose stringent criteria for the initial assembly step. For the diploid *F. pratensis* minimum overlap length (L) was set to 20% of the read length, and percent similarity for read overlap (S) was set to 99%. In the second step of the *de novo* assembly we re-assembled contigs from the first step to allow for the collapse of diverse alleles into single contigs. This was done by performing a second *de novo* assembly in CLC with the genotype specific contigs from step 1 as input, using the parameters L=80% and S = 95%.

References

Ergon, A., C. Fang, et al. (2006). "Quantitative trait loci controlling vernalisation requirement, heading time and number of panicles in meadow fescue (Festuca pratensis Huds.)." Theoretical and Applied Genetics **112**(2): 232-242.

Methods S2:

Sequencing, assembly and expression analyses of differential expressed transcripts between cold treated and non-cold treated *Lolium perenne* plants

Plant material for RNA isolation

Leaf material was collected at following time points: 0 hours, 2 days, 4 weeks, and 9 weeks of vernalization. Plants were grown for 2 months with simulated fall conditions (15°C and 8 hour day length) before initiation of vernalization treatment (6°C and 8 hour day length). Plant material was harvested between 9-10 am.

Sequencing

One biological replicate and three technical replicates were used in the sequencing process. The value of the Pearson's correlation coefficient for the three technical replicates was >0.99, thus all three technical replicates were pooled to aquire higher sequencing depth (Wang et al. 2010). A total of 68.852.859 reads with a length of 50 bp were generated on the Illumina Genome Analyzer II.

De novo assembly of reads

Reads from both leaf and enriched meristem were combined and subsequently assembled using Trinity software (Grabherr et al. 2011). The number and length of the assembled contigs are presented in the table below.

Analyses of differential expression

In order to assess the expression level, we used RSEM software (Li and Dewey 2011) to generate the raw count data for each assembled transcript**.** Sequence reads were mapped onto the assembled sequences, allowing up to one mismatch in the seed region (the first 25 bp) of the reads. More than 90% of reads were reported with at least one alignment across all time points and all samples.

DESeq software (Anders and Huber 2010) was used to assess differential expression between the non-vernalized and vernalized plants at different time points. The option involving no biological replicates identified a total of 720 differentially expressed transcripts across all time points.

References

Anders, S. & W. Huber (2010) Differential expression analysis for sequence count data. *Genome Biol,* 11**,** R106.

Grabherr, M. G., B. J. Haas, M. Yassour, J. Z. Levin, D. A. Thompson, I. Amit, X. Adiconis, L. Fan, R. Raychowdhury, Q. Zeng, Z. Chen, E. Mauceli, N. Hacohen, A. Gnirke, N. Rhind, F. di Palma, B. W. Birren, C. Nusbaum, K. Lindblad-Toh, N. Friedman & A. Regev (2011) Full-length transcriptome assembly from RNA-Seq data without a reference genome. *Nat Biotechnol,* 29**,** 644-52.

Li, B. & C. N. Dewey (2011) RSEM: accurate transcript quantification from RNA-Seq data with or without a reference genome. *BMC Bioinformatics,* 12**,** 323.

Wang, L., Z. Feng, X. Wang & X. Zhang (2010) DEGseq: an R package for identifying differentially expressed genes from RNA-seq data. *Bioinformatics,* 26**,** 136-8.

Table S1. Over-represented molecular function gene ontology (GO) terms in the low temperature induced genes subset. GO terms are grouped according to their GO slim group. Some terms are represented in multiple GO slim groups.

GO slim group	GO	GO description
Molecularc function (GO:0003674)	GO:0016209	Antioxidant activity
	GO:0004602	Glutathione peroxidase activity
DNA binding (GO:0003677)	GO:0003918	DNA topoisomerase (ATP-hydrolyzing) activity
RNA binding (GO:0003723)	GO:0003723	RNA binding
	GO:0030515	snoRNA binding
Catalytic activity (GO:0003824)	GO:0004812	Aminoacyl-tRNA ligase activity
	GO:0016875	Ligase activity, forming carbon-oxygen bonds
	GO:0004619	Phosphoglycerate mutase activity
	GO:0003756	Protein disulfide isomerase activity
	GO:0015036	Disulfide oxidoreductase activity
	GO:0004602	Glutathione peroxidase activity
	GO:0003883	CTP synthase activity
	GO:0015038	Glutathione disulfide oxidoreductase activity
	GO:0015035	Protein disulfide oxidoreductase activity
	GO:0003918	DNA topoisomerase (ATP-hydrolyzing) activity
	GO:0004133	Glycogen debranching enzyme activity
Structural molecule activity (GO:0005198)	GO:0003735	Structural constituent of ribosome
	GO:0017056	Structural constituent of nuclear pore
Transporter activity (GO:0005215)	GO:0008565	Protein transporter activity
	GO:0005384	Manganese ion transmembrane transporter activity
Binding (GO:0005488)	GO:0005507	Copper ion binding
	GO:0030170	Pyridoxal phosphate binding
	GO:0019842	Vitamin binding
	GO:0030247	Polysaccharide binding
Protein binding (GO:0005515)	GO:0019903	Protein phosphatase binding
	GO:0051082	Unfolded protein binding
Translation factor activity (GO:0008135)	GO:0003743	Translation initiation factor activity
	GO:0003746	Translation elongation factor activity
Lipid binding (GO:0008289)	GO:0005546	Phosphatidylinositol-4,5-bisphosphate binding
Transferase activity (GO:0016740)	GO:0004576	Oligosaccharyl transferase activity
	GO:0016760	Cellulose synthase (UDP-forming) activity
Hydrolase activity (GO:0016787)	GO:0004630	Phospholipase D activity
	GO:0008967	Phosphoglycolate phosphatase activity
	GO:0003918	DNA topoisomerase (ATP-hydrolyzing) activity
	GO:0016798	Hydrolase activity, acting on glycosyl bonds
Enzyme regulator activity (GO:0030234)	GO:0001671	ATPase activator activity
Carbohydrate binding (GO:0030246)	GO:0030247	Polysaccharide binding

Table S1. Under-represented molecular function gene ontology (GO) terms in the low temperature induced genes subset. GO terms are grouped according to their GO slim group. Some terms are represented in multiple GO slim groups.

GO slim group	**GO**	**Description**
DNA binding (GO:0003677)	GO:0003677	DNA binding
Binding (GO:0005488)	GO:0008270	Zinc ion binding
	GO:0020037	Heme binding
Kinase activity (GO:0016301)	GO:0004674	Protein serine/threonine kinase activity
	GO:0016301	Kinase activity
	GO:0004713	Protein tyrosine kinase activity
Transferase activity (GO:0016740)	GO:0016773	Phosphotransferase activity, alcohol group as acceptor
	GO:0004674	Protein serine/threonine kinase activity
	GO:0004713	Protein tyrosine kinase activity

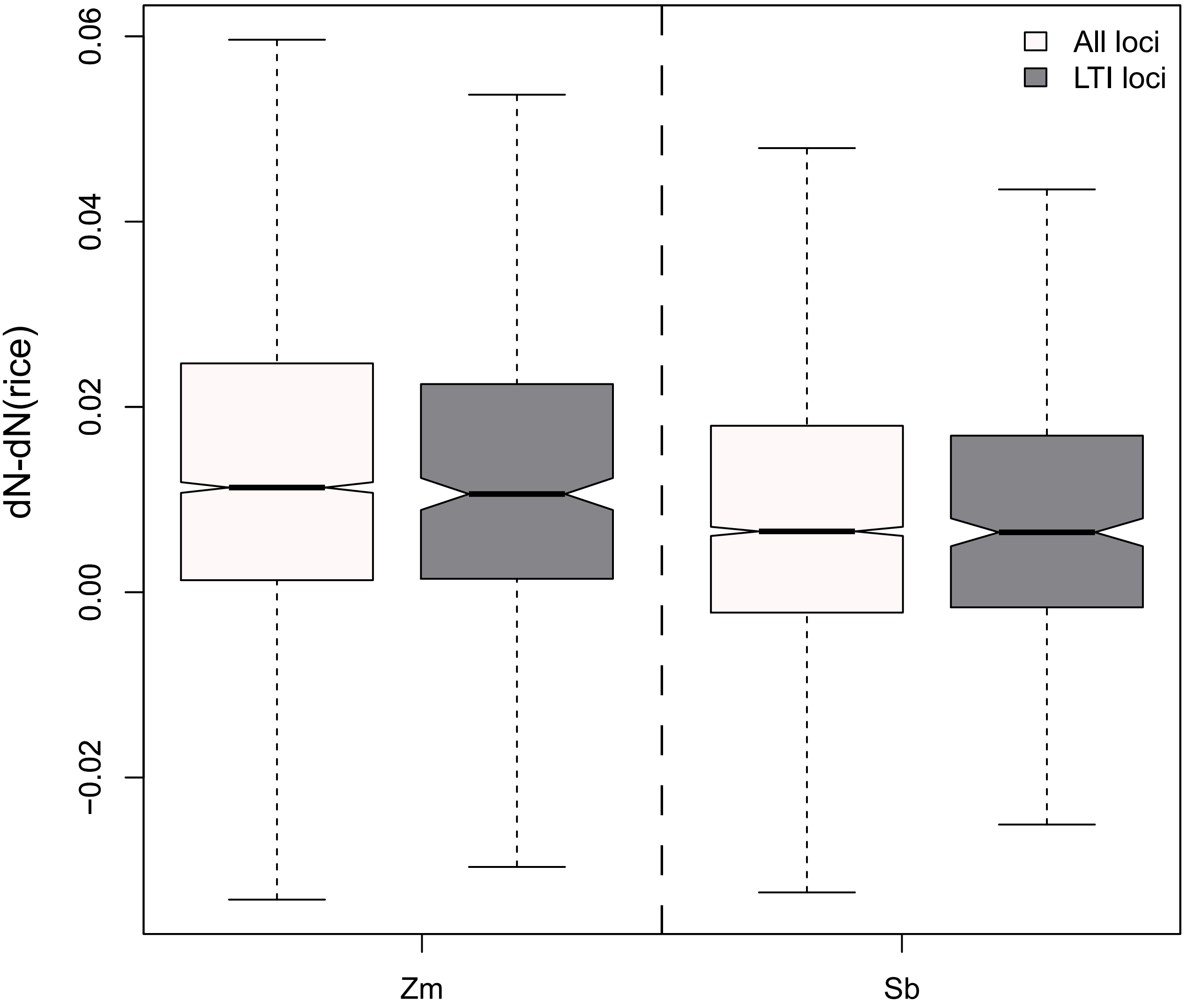

Figure S1. dN rate differences between maize-rice and sorghum-rice. Species abbreviations: Zm = maize, Sb = sorghum.
